# Utilization Patterns and Perceptions of a Spaced Repetition Flashcard Program, Anki, Among First-Year Medical Students

**DOI:** 10.7759/cureus.95674

**Published:** 2025-10-29

**Authors:** Hanna Nour, David M Harris

**Affiliations:** 1 Academic Affairs, University of Central Florida College of Medicine, Orlando, USA; 2 Medical Education, University of Central Florida College of Medicine, Orlando, USA

**Keywords:** anki flashcards, medical education, medical education resources, spaced repetition flashcards, third-party resources

## Abstract

Introduction: Medical students often turn to external resources to optimize their learning efficiency due to vast amounts of information. A popular resource is Anki (Ankitects Pty Ltd., New South Wales, Australia) a free, open-source flashcard program that leverages spaced repetition to enhance learning and retention. Studies have shown that spaced education enhances knowledge retention in medical education; however, few have specifically examined Anki's usage patterns and perceptions to identify potential interventions that could help enhance learning outcomes. The purpose of this study was to determine usage patterns and student perceptions of how they utilize Anki. Furthermore, this study looked at a deeper level of how students incorporated it into their lives outside of school.

Methods: First-year medical students at the University of Central Florida College of Medicine were recruited to participate in a survey. Deidentified demographic data were gathered, and the survey was open to both Anki and non-Anki users and incorporated question responses using the Likert scale. For Anki users, the survey examined their Anki usage structure, perceptions of Anki, and Anki in relation to life activities. Survey questions were created based on prior Anki experience and adapted from various topics on validated burnout surveys. Seventy-four percent of the first-year class participated in the survey. Quantitative analysis was performed using descriptive statistics and correlation tests on SPSS.

Results: Most students identified as Anki users (94%), emphasizing its role in medical education, and most users relied on pre-made cards (97.6%) and considered Anki as a key study strategy (62.6%). Most students preferred fill-in-the-blank cards (78%), 59% believed Anki boosted productivity compared to other study tools, and a substantial majority (87.8%) believed that Anki significantly contributes to their success in modules. Anki also affected “everyday” life activities. A majority (56.6%) reported multitasking with Anki during activities like exercising or eating. In addition, a Pearson correlation test revealed that the higher the percent study strategy of Anki, the stronger the perception of success (r= 0.621, p<0.001).

Conclusion: Most medical students use Anki as part of their study strategy. Faculty may be able to utilize these insights to optimize the implementation of Anki in medical education by helping students understand how to best utilize Anki’s best practices. Future research could further explore students’ reasons for how they use this resource.

## Introduction

Medical education is notorious for its immense challenges, demands, and ever-growing body of knowledge. Given the need to process vast amounts of information within short timeframes, medical students often turn to resources outside of the formal curriculum to optimize their learning. One popular resource is Anki (Ankitects Pty Ltd., New South Wales, Australia), a free, open-source flashcard program that leverages spaced repetition theory to enhance learning and retention [1]. Anki allows students to make their own flashcards or access pre-made flashcards or decks from classmates and third-party sources. Once the flashcards are acquired, students can use their desktop application, mobile application, or log on to Anki-Web from any device to study the flashcards. Students even pair Bluetooth remotes with the flashcard program to increase their efficiency. 

Anki is based on spaced repetition, which is a learning strategy based on the psychological spacing effect [2,3]. Spaced testing provides students with repeated exposure to information at varying time points, improving their long-term memory. Additionally, several studies have demonstrated that spaced education can enhance clinical knowledge retention in medical school, with increased usage being linked to higher scores on the United States Medical Licensing Examination Step 1 [4-6] and other standardized examinations [7]. These studies have explored the relationship between Anki usage and performance on medical exams, demonstrating its potential to boost academic success. However, there is limited research on the specific ways students use Anki to enhance their learning. This gap leaves questions about how study habits, frequency of use, and types of cards contribute to student outcomes. Understanding these usage patterns is crucial, especially considering findings from Kim et al., which reveal that although faculty generally have positive attitudes toward e-learning tools like Anki, various barriers, such as lack of training, time constraints, or uncertainty about how to integrate these tools into existing curricula, often prevent their incorporation into teaching strategies [8].

The purpose of this study was to determine usage patterns and student perceptions of how they utilize Anki. Furthermore, this study looked at a deeper level of how students incorporated it into their lives outside of school. The findings of this study will provide important data to faculty about the overall impact and influence of Anki on first-year medical students. This is critical as Anki can be time-consuming and has a large learning curve, which could affect how students perform overall and their well-being.

This paper was accepted as an abstract for the University of Southern California’s Innovations in Medical Education Online Conference (February 15-16, 2024) as an oral presentation.

## Materials and methods

Context of curriculum

The curriculum at the University of Central Florida College of Medicine consists of two years of pre-clerkship modules and two years of clinical courses. The first year consists of three basic science modules (Cell and Molecular Genetics, Structure and Function, and Health and Disease) as well as a year-long Practice of Medicine module and research module. Each annual class has about 120 students.

Survey and participants

This study was determined to be exempt from human subject research by the University of Central Florida’s (UCF) Institutional Review Board (IRB). First-year medical students (Class of 2026) at the UCF College of Medicine were recruited to participate in an online survey designed to explore their usage patterns and perceptions of Anki.​ Inclusion criteria included being enrolled at UCF as a first-year medical student. 

The survey was distributed through Qualtrics via email and GroupMe, beginning at the end of their first year and continuing into the early part of their second year. Participants were compensated with a $10 Amazon gift card through email. Survey questions were created based on the author’s Anki experience and adapted based on surveys from similar research on Anki usage [6,8]. Survey questions were made based on prior Anki experience and measures of respective domains. Survey questions were content validated by the authors of the study prior to release. We also viewed validated external surveys and based questions on those to aid in validation. 

Deidentified demographic data, including age, sex, academic path, and undergraduate majors, in addition to pertinent survey questions, were gathered. The survey was open to both Anki users and non-Anki users and was mostly formatted using a Likert scale. For Anki users, the survey examined their Anki usage structure, perceptions of Anki, and Anki in relation to burnout and wellness. For non-Anki users, the survey examined if they have ever used Anki and, if so, why they discontinued usage. The data were anonymized and analyzed through IBM SPSS Statistics software v. 30 (IBM Corp., Armonk, NY), and quantitative data analysis was performed using descriptive statistics and Pearson correlation tests [9].

## Results

Demographics

The first questions on the survey served to identify Anki users from non-Anki users, as well as other demographic data. The response rate of the survey was 74.1% as 89 of 120 possible students participated in the study (Table 1). The majority of survey takers were Anki users (94%). The median age of the participants was 24 (range of 21-34) years of age. The gender distribution of Anki users was almost equal, and most student responses indicated that they had 0 or 1 gap years (71%). 

**Table 1:** Demographics and Percentage of Anki Users

Total Participants	Frequency (n)	%
Anki users	84	94%
Non-Anki users	5	6%
Age		
Median age	24	
Minimum age	21	
Maximum age	34	
Gender Distribution (Anki Users)	84	
Female	43	51%
Male	41	49%
Gap Year Experience (Prior to Matriculation)		
Traditional student (0 gap years)	23	27%
1 gap year	37	44%
2 gap years	14	17%
3+ gap years	10	12%

Frequency and adoption

The first set of questions aimed to see how many medical students used Anki prior to medical school and how often they used it during medical school. Non-Anki users were not asked additional questions, as the Anki users continued to the survey. As can be seen in Table 2, almost half (48.2%) of the students had not used Anki before medical school. Students used it extensively, as 67.9% of students used Anki at least five days a week. The majority of students did not use it after completing a module, as 75% of students never or rarely kept up with daily reviews. These data show that a high percentage of students use Anki at least five days a week, but do not use it to re-study previous courses/modules.

**Table 2:** Anki Frequency and Use ^†^denotes only 83 participants answered this question

Topics	Frequency (n)
Had not used Anki before medical school^†^	40
Use Anki at least 5 days a week	56
Students never/rarely keep up with daily reviews from previous modules	63

Anki strategy and perceptions

The next set of survey questions aimed to understand how students use Anki and their perceptions of how it affects their success (Table 3). Students believe that Anki increases their productivity compared to other study tools. Another critical aspect of use is whether students develop their own decks or utilize pre-made decks. Around 97% of students responded that they utilized pre-made Anki cards and decks from third-party sources. Anki cards also come in different styles or formats, such as fill-in-the-blank and open-ended questions. Students (78.3%) also preferred fill-in-the-blank cards vs other formats in the design of their cards. 

**Table 3:** Anki Strategies and Perceptions of Success ^†^ out of 83 students as 1 student did not answer this question, ^††^ out of 82 students as 2 students did not answer this question, **=denotes p<.001 The higher the percentage of Anki study strategy, the stronger the perception of success (r=.621**), The higher the average daily card #, the higher perception of success. (r=.330**)

Topics	Frequency (n)	%
Believe Anki boosts productivity compared to other study tools^†^	49	59.0%
Utilize pre-made Anki cards^†^	81	97.6%
Prefer fill-in-the-blank cards versus other formats	65	78.3%
Believe that Anki significantly contributes to their success in modules^††^	72	87.8%
Anki is at least 50% of their study strategy†	52	62.6%

Most students believe that Anki contributes to their success in the modules. It is important to recognize that students (62.6%) consider Anki at least 50% of their study strategy. Furthermore, the higher percentage of using Anki as their study strategy, the stronger the perception of success (r=0.621, p<0.001). Additionally, the higher the average daily card number, the higher the perception of success (r=0.330, p<0.001). 

Anki usage and lifestyle

The flexibility and accessibility of the Anki flashcard system were evaluated with the next set of questions (Table 4). The survey was designed to understand how Anki is utilized in relation to “everyday” activities and lifestyle. More than half of students reported that they used Anki while they were exercising or eating (56.6%). An advantage of Anki is that it can be done during exercise, as almost ¾ of respondents reported using Anki while exercising. On the other hand, over half of the students have skipped exercise to do Anki cards instead (52.5%). Importantly, other lifestyle factors may be affected as students have lost sleep to Anki (34.5%) or forgotten to eat (36.9%). These data show that while Anki may be used during exercise by many students, there are also effects on other important lifestyle factors, such as sleep and eating.

**Table 4:** Anki Effects on Lifestyle and "Everyday" Activities ^†^out of 83 students, as one student did not answer the question

Topics	Frequency (n)	%
Report multitasking with Anki during activities like exercising or eating^†^	47	56.6%
Have skipped exercising due to Anki	44	52.5%
Can exercise and do Anki at the same time^†^	62	74.7%
Have done Anki before going to sleep	57	68.3%
Have lost sleep due to Anki	29	34.5%
Have forgotten to eat due to Anki	31	36.9%

Anki and emotional impact

The next set of survey questions aimed to elaborate on various feelings associated with the use of Anki (Table 5). Interestingly, although the majority of students utilize Anki, there were many students who found Anki overwhelming (82.1%), making them feel anxious (67.9%). A large majority of students felt accomplished when they completed their daily reviews (95.1%) and felt frustrated if they did not complete them (68.3%). These data show that Anki usage does have an emotional impact on users, which could be perceived as positive or negative.

**Table 5:** Anki Effects on Feelings

Topics	Frequency (n)	%
Find Anki overwhelming to various extents	69	82.1%
Feels that Anki makes them feel anxious to various extents	56	67.9%
Feels frustrated when they do not complete their daily reviews	57	68.3%
Feels accomplished/satisfied when they complete their daily reviews	78	95.1%

## Discussion

The current study provides important findings on what features of Anki students utilize, how it is used in’ “everyday” life of medical students, and how it can affect them emotionally and personally. The major findings of the study include that almost all students utilize Anki daily, particularly pre-made decks from other students and third-party resources. Other important findings include that students only use it for one module and not across modules or the academic year, and that Anki’s use can affect the lifestyles of students in a potentially positive and negative manner. An interesting and novel finding from the current study is how students utilize Anki while doing everyday tasks such as eating and exercise, as well as how it may affect sleep. The significance of the findings of this study can help faculty understand the broader implications of how students utilize Anki and how it can affect them psychologically.

The current study showed that 94% of the participants in the survey were Anki users. This percentage is higher than what has been reported in the literature in a recent systematic and meta-analysis by Kann et al [10]. The manuscript showed a range of the percentage of Anki usage from 22-70% of participants in the studies [5, 11-13]. Interestingly, even at the University of Central Florida, we previously reported lower percentages of students using Anki in previous years and studies compared to the current study [7,14]. This could be due to a national trend across medical schools. Recent reports from the Boonshoft School of Medicine (SOM) reported 60% usage among their students, the Kirk Kerkorian SOM reported 57.7% and a national survey of 560 medical students across 102 US medical schools reports 68.3% Anki usage, showing that the University of Central Florida College of Medicine is above the national average usage [7,15,16]. This could be a result of many non-Anki users not participating in the survey, although there was a robust response rate of 78%. It could be due to only surveying first-year medical students in the current study, as there is limited literature on how the needs of students change during the progression of medical education, including the use of external resources. Additionally, there could be influences of the curriculum, formal and “hidden,” that affect usage, such as alignment with assessments or advice of upper classmates. 

 A recent scoping review by Barrison et al. showed that most published papers on the use of electronic flashcards focused on the utilization and associated outcomes, such as test performance [17]. Fewer studies focused on the development of electronic flashcards and delivery methods. The current study adds to this literature by showing strongly that almost all Anki users prefer using pre-made decks instead of writing and developing their own Anki decks and cards. Some of the educational implications are that students bypass critical thinking skill development or the productive struggle by using Anki. Faculty could use their experience to help students navigate how best to use Anki as a recall or memory tool, instead of a deeper learning method. In the 64 studies utilized in the scoping review, only five used crowd-sourced or learner-created cards or Anki decks. A study by Loving et al. found that 80% of participants found Anki cards created by near peers to be helpful compared to only 29% finding third-party resources created, and 48% of self-made cards [18]. Interestingly, although not directly asked in the current study, many of our students expressed that they usually use cards made by upperclassmen, supporting the findings by Loving. This is most likely due to alignment with the school’s curriculum. 

To our knowledge, the current study is one of the first to research how students incorporate Anki into their daily lives in greater detail. Our data shows that one of the advantages of Anki may be the ability to multitask with other activities. We show that about three-fourths of students do Anki while they are exercising, which is possible using walking treadmills and study stations in our school. A study by Wothe et al. observed improved quality of sleep with Anki usage, but no outcomes on well-being outcomes such as stress and leisure time [19]. Our study shows that a percentage of students (about 33%) have lost sleep or forgotten to eat as a result of using Anki. Faculty should be made aware of many of these findings, as this may lead to intervention efforts to help students utilize Anki as a tool correctly. This suggests that lifestyle effects need to be considered in the usage and implementation of Anki as a resource. 

This study has several limitations that may impact its findings. First, it focused exclusively on medical students at the end of their first year, which restricts the analysis over time and raises the possibility that results could differ across subsequent school years. Additionally, the reliance on self-reported data through surveys introduces the potential for bias, as responses may not accurately reflect true usage or perceptions. Although 74% (89 out of 120) of first-year medical students participated in the survey, response bias may have influenced the results; students with a more favorable view of Anki might have been more likely to complete it. We only had five non-Anki users participate in this study, which limited our ability to be able to perform comparison studies. Finally, conducting the study at a single medical school limits the generalizability of the findings to other institutions or educational settings. Future studies could focus on comparing usage across schools or at various time points in a student's career. 

## Conclusions

In conclusion, this study underscores the widespread use of Anki among first-year medical students, indicating its importance as a learning resource in their academic journey. The insights gained from this research could prove invaluable for faculty members seeking to enhance educational strategies within medical curricula. Additionally, this data could help prepare faculty on how to best help students utilize best practices such as spaced repetition. Future research could delve deeper into why certain methods of flashcard delivery are preferred (fill in the blank) and if there are differences between that type and others. Additionally, how Anki affects everyday life activities, positively and negatively, needs to be considered. Ultimately, this data could serve to help faculty transition to coaching students through different study strategies.

## Appendices

Survey for Anki users

Demographics

1. What is your age? (enter a numerical value)

• Input answer

2. What is your sex?

• Male

• Female

3. What is your gender?

• Woman

• Man

•Non-binary/other

4. Which answer choice best fits your academic path before after graduating undergrad and before matriculating into medical school?

• Traditional student (0 gap years, matriculated directly into medical school after graduating undergrad)

• 1 gap year

• 2 gap years

• 3+ gap years

5. Which best fits your undergraduate/graduate major(s)/minor(s)? Select all that apply:

• Science/Technology/Engineering/Mathematics (STEM)

• Social science/other

Survey Opening

1. Is Anki a part of your study strategy?

• Yes

• No (I do not use it at all)

2. When did you first try Anki?

• Before medical school

• During HB1 (Cellular Function and Medical Genetics)

• During HB2 (Structure and Function)

• During HB3 (Health and Disease)

• During C1 (Psychosocial Issues in Healthcare)

• During S1 (Hematology and Oncology)

3. How often do you use Anki in a week?

• Everyday

• 5-6 days per week

•3-4 days per week

• 1-2 days per week

4. During the school year, do you keep up with your daily reviews (cards with green number) for the current module?

• Never

• Rarely

• Sometimes

• Often

• Always

5. During the school year, do you keep up with your daily reviews (cards with green number) for the previous modules?

• Never

• Rarely

• Sometimes

• Often

• Always

6. If so, which subjects?

• Genetics

• Biochemistry

• Anatomy

• Physiology

• Embryology

• Histology

• Microbiology

•Pharmacology

• Immunology

• Hematology/Oncology

7. On average, how many hours do you use Anki per day?

•  Input number

8. What is your average number of cards per day in the last year? Instructions for finding this Anki statistic are below.

• Input number

a. Open Anki desktop app

b. Press the letter “T” (this will open a Statistics tab).

c. The top of the Statistics page will have the options:

• Deck

• Collection

• Search

• last 12 months

• all history

d. Make sure that “Collection” option is selected”

e. Scroll down to the bar graph labeled as “Reviews”

f. Below the“Reviews” word, there will be the following answer choices:

•Time

• 1 month

• 3 months

• 1 year

g. Make sure the Time is UNSELECTED (blank)

h. Select the 1-year option.

i. The following statistics will be below the bar graph:

• Days Studied:

• Total:

• Average for days studied:

• Average over period:

j. Make note of the number for “Average for days studied”

k. Input this number into the survey

6. Do you create your own cards, use pre-made cards, or a mixture?

• I create my own cards

• I use pre-made cards (from classmates, external sources, etc.)

• I use pre-made cards AND make my own

7. If you make your own cards, do you use lecture material and/or outside resources? Select all that apply:

• Lecture material

• Outside resources

• Lecture material AND outside resources

8. How do you use Anki in relation to lecture?

• Lecture first and then Anki

• Anki first and then lecture

• Only Anki (I do not watch lecture)

9. How do you use Anki in relation to outside material (secondary resources/dark curriculum)?   

• Outside material first and then Anki

• Anki first and then outside material

• Only Anki (I do not use outside material)

10. Which subjects do you use Anki for? (Check all that apply)

•Genetics

• Biochemistry

•Anatomy

• Physiology

• Embryology

• Histology

• Microbiology

• Pharmacology

• Immunology

• Hematology/Oncology

11. What percentage of your study strategies does Anki take up?

• 0% Anki, 100% other

• 10% Anki, 90% other

• 20% Anki, 80% other

• 30% Anki, 70% other

• 40% Anki, 60% other

• 50% Anki, 50% other

• 60% Anki, 40% other

• 70% Anki, 30% other

• 80% Anki, 20% other

• 90% Anki, 10% other

• 100% Anki, 0% other

12. Choose the option that BEST matches how you navigate through MOST flashcards

• I have to remember every word of the card to mark it as correct (good)

• I do not have to remember every word on the card, rather, just the concept to mark it as correct

• I click through cards and read the material rather than answering each card

13. When using Anki, how often did you do each of the following?

Never (1)

Sometimes (2)

About half the time (3)

Most of the time (4)

Always (5)

Press "Again" if the card was not perfectly recalled (1); Took time to understand the concepts behind the card and not just use rote memorization (2)

14. Which style of cards do you prefer the most?

•Basic

• Cloze deletions (fill in the blank)

• Image occlusion (blocked out image)

• Other

§ *include a screenshot of the different types

15. When a deck is primarily consists of basic cards as opposed to Cloze deletions cards:

• I feel less stressed

• I feel more stressed

• No change

16. When a deck is primarily consists of cloze deletions cards as opposed to basic cards

• I feel less stressed

• I feel more stressed

• No change

Perceptions of Anki

1. I believe Anki significantly contributes to my success in the modules

• Strongly Agree

• Agree

• Disagree

• Strongly Disagree

2. I find Anki overwhelming.

• Never

• Rarely

• Sometimes

• Often

• Always

3. Anki makes me feel anxious

• Never

• Rarely

• Sometimes

• Often

• Always

4. I feel frustrated when I do not finish my daily reviews

• Strongly Agree

• Agree

• Disagree

• Strongly Disagree

5. I feel accomplished/satisfied when I finish my daily reviews

• Strongly Agree

• Agree

• Disagree

• Strongly Disagree

6. Rate your level of anxiety for an examination (test/quiz) when BEFORE completing the relevant cards

• High

• Moderate

• Low

• None

7. Rate your level of anxiety for an examination (test/quiz) when AFTER completing the relevant cards

• High

• Moderate

• Low

• None

8. Rate your level of confidence for an examination (test/quiz) when BEFORE completing the relevant cards

• High

• Moderate

• Low

• None

9. Rate your level of confidence for an examination (test/quiz) when AFTER completing the relevant cards

• High

• Moderate

• Low

• None

Anki and Wellness

1. I use Anki while I do other activities (exercising, eating, stopped at a red light, watching a show)

• Never

• Rarely

• Sometimes

• Often

• Always

• N/A

2. Anki makes me feel more productive in comparison to other study tools because I am able to combine it with other activities (exercising, eating, stopped at a red light, watching a show)

• Strongly Agree

• Agree

• Neutral

• Disagree

• Strongly Disagree

3. I have lost sleep due to Anki

• Never

• Rarely

• Sometimes

• Often

4. I have skipped exercising due to Anki

• Never

• Rarely

• Sometimes

• Often

• Always

5. I have forgotten to eat due to Anki

• Never

• Rarely

• Sometimes

• Often

• Always

6. I can exercise and do Anki at the same time (treadmill desk, going for a walk)

• Strongly Agree

• Agree

• Disagree

• Strongly Disagree

7. I do Anki before going to sleep

• Never

• Rarely

• Sometimes

• Often

• Always
